# Systemic Sclerosis: A Key Model of Endothelial Dysfunction

**DOI:** 10.3390/biomedicines13071771

**Published:** 2025-07-19

**Authors:** Vincenzo Zaccone, Lorenzo Falsetti, Silvia Contegiacomo, Serena Cataldi, Devis Benfaremo, Gianluca Moroncini

**Affiliations:** 1 PhD Course in Human Health, Marche Polytechnic University, 60126 Ancona, Italy; vincenzozaccone@gmail.com; 2 Clinica Medica, Department of Clinical and Molecular Sciences, Marche Polytechnic University, 60126 Ancona, Italy; l.falsetti@staff.univpm.it (L.F.); silviacontegiacomo@gmail.com (S.C.); g.moroncini@univpm.it (G.M.); 3 Department of Pediatrics, Marche University Hospital, 60126 Ancona, Italy; cataldiserena@gmail.com

**Keywords:** systemic sclerosis, SSc, endothelial dysfunction, endothelial-to-mesenchymal transition

## Abstract

Systemic sclerosis (SSc) is a heterogeneous disease characterized by vascular alterations, immune dysregulation, and fibrosis. Solid evidence supports the hypothesis that endothelial dysfunction is the key player in SSc vascular injury and a critical factor concurring to the initiation of SSc pathogenesis. This narrative review reports on persistent endothelial dysfunction, resulting from oxidative stress, autoimmunity, and impaired vascular repair, in the course of SSc, and how it can trigger and sustain fibrotic remodeling of various organs. In this paper, we also analyze the impact on SSc of impaired angiogenesis and vasculogenesis, diminished endothelial progenitor cell function, and endothelial-to-mesenchymal transition, which can collectively disrupt vascular homeostasis and promote myofibroblast activation. These pathologic events underlie the hallmark clinical manifestations, i.e., Raynaud’s phenomenon, digital ulcers, pulmonary arterial hypertension, and scleroderma renal crisis. The review highlights how recognizing SSc as a paradigm of systemic endothelial dysfunction may reframe our understanding of its physiopathology, modify current therapeutic strategies, and unveil new therapeutic targets.

## 1. Systemic Sclerosis and Endothelial Dysfunction: Background and Rationale

Systemic sclerosis (SSc) is a connective tissue disease characterized by vascular abnormalities, immune dysregulation, and progressive fibrosis. While fibrosis of the skin and internal organs is a later clinical feature, the earliest clinical event is Raynaud’s phenomenon, which strongly suggests endothelial dysfunction and microvascular alterations as early drivers of disease onset and progression [1]. The vascular component of SSc is complex and involves persistent endothelial activation, defective angiogenesis, and dysregulated vasculogenesis. These factors lead to chronic ischemia of peripheral limbs with increased susceptibility to severe complications, such as digital ulcers, and possibly also to pulmonary arterial hypertension (PAH) and scleroderma renal crisis [2].

Endothelial dysfunction is defined by an imbalance in endothelial homeostasis that leads to impaired vasodilation, increased permeability, pro-inflammatory activation, and a pro-thrombotic state. In the context of SSc, multiple studies have reported alterations in endothelial function, including decreased nitric oxide (NO) bioavailability, heightened endothelin-1 (ET-1) levels, and increased expression of adhesion molecules such as vascular cell adhesion molecule-1 (VCAM-1) and E-selectin. These changes contribute to abnormal regulation of vascular tone, endothelial apoptosis, and obliterative arteriolopathy with subsequent capillary rarefaction, which are hallmarks of the disease [3].

Beyond microvascular injury, recent research suggests that macrovascular involvement may also play a role in SSc pathophysiology. Increased carotid intima-media thickness (IMT), arterial stiffness, and early atherosclerotic changes have been observed in SSc patients; however, the precise mechanisms linking micro- and macrovascular dysfunction remain largely unknown [4]. Flow-mediated dilation (FMD) and circulating endothelial biomarkers offer additional evidence of systemic endothelial impairment in SSc, reinforcing the hypothesis that endothelial dysfunction is a primary driver of disease [5].

In this narrative review, we aim to examine the literature on endothelial dysfunction in SSc and discuss the mechanistic underpinnings of vascular abnormalities, their clinical implications, and potential therapeutic strategies. Understanding endothelial dysfunction as a core feature of SSc may provide insights into broader vascular pathologies associated with autoimmune and fibrotic diseases.

## 2. Materials and Methods

We searched PubMed/MEDLINE for case reports, reviews, and original research articles from 1 January 1992, to 1 May 2025. We used MeSH major terms and considered the following: “Scleroderma, Systemic” [Mesh] or “Scleroderma, Diffuse” [Mesh] in association with “Endothelium” [Mesh], “Endothelium, Vascular” [Mesh], “Vasodilation” [Mesh], “Nitric Oxide” [Mesh], “Endothelium-Dependent Relaxing Factors” [Mesh], “Angiogenesis” [Mesh], or “Endothelial-Mesenchymal Transition” [Mesh]. The group of reviewers favored the inclusion of articles from the past 5 years, although they did not exclude highly cited older reports. The reference lists of articles identified by this search strategy were also reviewed, and the working group selected relevant references.

## 3. Endothelial Cell Damage

Vascular endothelial cell injury represents a pivotal event in the pathogenesis of SSc, setting the stage for the development of microvascular dysfunction, progressive ischemia, and tissue fibrosis [1]. The process is likely triggered by an initial insult to the endothelial cells, followed by a maladaptive vascular response involving intimal proliferation, obliterative arteriolopathy, and capillary rarefaction. The etiological factors contributing to endothelial damage in SSc are yet to be identified, possibly involving viral agents, exposome, autoimmune mechanisms, and oxidative stress, all of which may interact to perpetuate the vascular injury [2].

The immune system plays a central role in endothelial damage, with innate and adaptive components contributing to endothelial cell apoptosis and dysfunction. In the early stages of SSc, perivascular infiltration by mononuclear lymphocytes, predominantly CD8+ cytotoxic T cells, has been identified in affected tissues. These immune cells interact with endothelial cells expressing Human Leukocyte Antigen (HLA) class I and II molecules, triggering apoptotic cascades [3]. Furthermore, autoantibodies directed against endothelial cell antigens (AECAs) have been detected in many patients and implicated in endothelial cytotoxicity via complement-dependent/Fas-independent apoptotic mechanisms [4]. In vitro models have demonstrated that AECAs induce endothelial cell activation, upregulating endothelin-1 expression and promoting a pro-inflammatory endothelial phenotype [5]. These findings support the hypothesis that autoimmunity contributes directly to vascular dysfunction in SSc.

Viral agents have also been suggested as possible triggers of endothelial injury, with several viruses identified in plasma and tissues of SSc patients [6]. Human cytomegalovirus (HCMV) has been implicated based on its capacity to target and infect endothelial cells directly and elicit an autoimmune response through molecular mimicry [7,8]. The presence of anti-HCMV antibodies cross-reacting with endothelial cell antigens suggests a mechanism by which viral proteins, such as UL94, may drive endothelial apoptosis [9]. Similarly, Epstein–Barr virus (EBV) has been shown to infect endothelial cells, leading to aberrant activation of toll-like receptor 9 (TLR9) and type I interferon pathways, which may compromise endothelial integrity [10]. Parvovirus B19 has also been implicated, as it can upregulate tumor necrosis factor-alpha (TNF-α) expression in endothelial cells, exacerbating endothelial activation and apoptosis [11].

Another major contributor to endothelial injury in SSc may be oxidative stress, which arises from repetitive ischemia–reperfusion cycles in Raynaud’s phenomenon. The generation of reactive oxygen species (ROS), including superoxide anions, hydroxyl radicals, and hydrogen peroxide, may lead to endothelial dysfunction by disrupting NO homeostasis. The reduction in bioavailable NO, a critical vasodilatory molecule, causes vasoconstriction and platelet aggregation. ROS also impair endothelial anticoagulant functions by inhibiting the release of prostacyclin, tissue plasminogen activator, and heparan sulfate, thereby promoting a pro-coagulant endothelial state. The cumulative impact of these oxidative insults is endothelial death, which accelerates capillary loss and exacerbates arteriolar occlusion [12].

Recent mechanistic studies have shed light on the complex role of IL-17A and related cytokines in SSc-associated vascular dysfunction. IL-17A exerts pro-inflammatory and pleiotropic effects on vascular smooth muscle cells, endothelial cells, and fibroblasts, and is implicated in vascular damage in SSc [13]. Importantly, IL-17A levels and IL-17+ lymphocyte infiltration are increased in SSc patients, promoting chemokine and adhesion molecule expression in endothelial cells and enhancing T cell adhesion via the ERK signaling pathway [14]. Beyond its broad range of actions, IL-17A has been shown to synergize with cytokines such as TNF-α and IL-1β. These synergistic interactions enhance the production of IL-6 and CXCL8 in fibroblasts and endothelial cells, thereby amplifying inflammation. Moreover, a recent study showed that targeting IL-17A could effectively modulate the activity of these cytokines and significantly influence the expression of molecules elevated in SSc patients at high risk for pulmonary arterial hypertension (PAH) [15].

Circulating biomarkers of endothelial damage provide additional evidence of widespread endothelial dysfunction in SSc. Increased serum concentrations of von Willebrand factor (vWF), soluble Junctional Adhesion Molecule-1 (sJAM-1), and endothelin-1 are consistent with sustained endothelial activation and vascular injury [16]. Immune complex deposition further amplifies endothelial activation, inducing the expression of interleukin-6 (IL-6), intercellular adhesion molecule-1 (ICAM-1), and transforming growth factor-beta (TGF-β1), which collectively drive vasoconstriction, inflammation, and fibrosis [17].

Recent high-resolution transcriptomic and proteomic analyses have further characterized the cellular complexity and molecular dysfunction of endothelial cells in SSc, offering deeper mechanistic insights into vascular injury.

The study by Huang et al. utilized single-cell RNA sequencing and chromatin analysis to investigate EC dysfunction in SSc [18]. Their findings revealed increased apoptosis-related gene expression and elevated proangiogenic activities in specific EC subpopulations, such as tip and proliferating ECs. Altered intercellular signaling networks were linked to SSc pathology, including skin fibrosis and digital ulcers. The study identified ETS transcription factors (ELK4, ERF, and ETS1) as key regulators of EC apoptosis and angiogenesis, suggesting potential therapeutic targets for addressing SSc-related vascular complications.

Renaud et al. compared the transcriptome of pericytes from SSc-PF lungs to pericytes from normal lungs, identifying prostaglandin, PI3K-AKT, calcium, and vascular remodeling signaling pathways among those enriched in SSc [19]. The transcriptomic signature of SSc lung pericytes suggested that they promote vascular dysfunction and contribute to the loss of protection against lung inflammation and fibrosis.

Using sera from SSc patients, Chepy et al. demonstrated that purified IgG from patients with SSc can modify EC proteome and transcriptome according to anti-nuclear antibody specificity [20]. In particular, IgG from ATA+ patients induced the most singular proteomic profile in EC, characterized by VEGFA and VEGFR2 signaling enrichment in up- and downregulated proteins, while VE-cadherin was overexpressed in EC in the presence of IgG ATA+ in both cohorts. IgG from ACA+ patients induced DEP enriched in VEGFA and VEGFR2 signaling in derivation cohort but was not retrieved in validation cohort.

Using spatial proteomics, Rigau et al. unraveled the heterogeneity of vascular cells in control individuals and patients with SSc [21], identifying CD34+ αSMA+ CD31+ ECs as a novel endothelial cell population that is increased in patients with SSc, expresses markers for endothelial-to-mesenchymal transition, and is located in close proximity to immune cells and myofibroblasts. CD34+ αSMA+ CD31+ EC counts were associated with clinical outcomes of progressive fibrotic remodeling, thus providing a novel cellular correlate for the crosstalk of vasculopathy and fibrosis.

The endothelial glycocalyx (EG) is a mesh-like layer covering the surface of ECs, composed of membrane-bound molecules such as proteoglycans and glycoproteins [22]. It plays a vital role in protecting ECs, mediating shear stress, facilitating nitric oxide production, and housing vascular protective enzymes and anticoagulant factors. Additionally, the EG regulates inflammation by controlling leukocyte adhesion and binding inflammatory mediators like chemokines, cytokines, and growth factors, thereby maintaining endothelial integrity. It also acts as a passive barrier, preventing protein and fluid leakage from capillaries and thus reducing edema. Functionally linked to the cell membrane and cytoskeleton, the EG is dynamic but can be damaged during ischemia/reperfusion injury due to EG-bound enzymes like xanthine oxidoreductase and superoxide dismutase [23].

Alterations in EG structure and function have been documented in various conditions, including sepsis, diabetes, obesity, cardiovascular disease, renal failure, and hypercholesterolemia [24]. Although data on SSc are limited, EG dysfunction in SSc likely impairs endothelial barrier integrity, reduces anticoagulant and anti-adhesive functions, and disrupts shear stress-induced nitric oxide release, compromising vascular tone regulation [25].

In SSc, sublingual microvessels show reduced total and perfused microvascular density, lower red blood cell (RBC) fraction, increased perfused boundary region (PBR)—indicating EG dysfunction—and decreased EG thickness, with an inverse correlation between PBR and EG thickness [26]. EG abnormalities necessitate RBC conformational changes to navigate microvessels and are linked to impaired endothelium-dependent vasodilation. Perfused microvascular density and RBC fraction are diminished across all microvessel sizes, highlighting a connection between microvascular health and EG integrity. Since the EG is crucial for vascular homeostasis and permeability, these findings may have clinical relevance in early SSc, a phase characterized by finger edema and capillary leakage.

## 4. Impaired Vasculogenesis

Vasculogenesis, i.e., the generation of new blood vessels from endothelial progenitor cells (EPCs), is critically impaired in SSc, further contributing to progressive vascular dysfunction [27]. EPCs, identified by surface markers such as CD34, CD133, and VEGFR2 (CD309), are relevant players in postnatal vascular repair and homeostasis [28]. Multiple studies have demonstrated a significant reduction in circulating EPCs in SSc patients compared to healthy controls, and its association with the presence of severe vascular manifestations, such as digital ulcers and pulmonary arterial hypertension (PAH) [2,29]. The depletion of EPCs is an independent predictor of the development of new digital ulcers and is associated with late-stage capillary abnormalities on nailfold capillaroscopy [30].

Beyond numerical deficiency, SSc-derived EPCs are also functionally impaired. In vitro experiments have demonstrated they are less able to differentiate into mature endothelial cells in response to pro-angiogenic factors, such as vascular endothelial growth factor (VEGF) and fibroblast growth factor-2 (FGF-2) [31]. Similarly, in vivo models of neovascularization, including murine tumor assays, have confirmed an impaired vasculogenic capacity in SSc-EPCs [32]. Mechanistically, this defect has been attributed to intrinsic alterations within the bone marrow microenvironment. The bone marrow in SSc displays pronounced fibrosis and a concomitant reduction in microvascular density, which impairs EPC mobilization and differentiation [33]. Additionally, circulating pentraxin-3, a recognized inhibitor of EPC differentiation through FGF2 suppression, is elevated in SSc patients, and inversely correlates with EPC counts [34].

Recent findings suggest that EPC dysfunction in SSc may also involve immune-mediated apoptosis. Studies have reported that EPCs in circulation are susceptible to immune attacks, with SSc patient sera inducing apoptosis via the Akt-FOXO3a-Bim pathway [35]. Furthermore, EPCs appear to be functionally impaired before their release from the bone marrow, as they fail to differentiate even under long-term culture conditions with pro-angiogenic stimuli [36].

The role of EPC subpopulations in SSc pathogenesis is gaining increasing attention. A specific subset, CD34+ CD133+ VEGFR3+ lymphatic EPCs, is particularly reduced in patients with active digital ulcers, suggesting possible involvement of lymphatic dysfunction [37]. Conversely, monocytic EPCs, a subset of hematopoietic progenitors with pro-angiogenic properties, are paradoxically increased in SSc [38]. However, these cells may contribute to fibrosis rather than vascular repair, as they express profibrotic mediators and are recruited to affected tissues in response to chemokine signaling, such as monocyte chemoattractant protein-1 (or C-C motif chemokine ligand 2: CCL2) and stromal cell-derived factor-1 (SDF-1) [39].

These findings highlight the multifaceted nature of EPC dysfunction in SSc. The interplay between numerical deficiency, impaired differentiation, bone marrow abnormalities, and immune-mediated apoptosis underlies the defective vasculogenesis observed in SSc, contributing to vascular rarefaction and chronic ischemia. Further research is needed to elucidate the precise mechanisms governing EPC dysfunction and explore potential therapeutic strategies to restore vasculogenesis in SSc patients.

## 5. Impaired Angiogenesis

Angiogenesis, the physiological process of new blood vessel formation from preexisting vasculature, is also dysregulated in SSc [2]. Despite the upregulation of multiple pro-angiogenic factors, including VEGF, FGF-2, and platelet-derived growth factor (PDGF), the expected neovascularization response is impaired, leading to progressive microvascular rarefaction and chronic tissue ischemia. This paradoxical finding suggests a complex dysfunction at multiple levels of the angiogenic signaling cascade.

Endothelial cells in SSc display intrinsic functional abnormalities contributing to their impaired angiogenic response. One of the key mechanisms involves a deficiency in endothelial nitric oxide synthase (eNOS), which results in reduced nitric oxide (NO) production and blunted endothelial migration in response to pro-angiogenic stimuli [40]. Additionally, the VEGF/VEGFR-2 signaling axis, essential for angiogenesis, appears to be disrupted. While circulating VEGF levels are elevated in SSc, the predominant upregulated isoform is VEGF165b, an anti-angiogenic variant that competitively inhibits VEGF165 binding to VEGFR-2, leading to insufficient downstream tyrosine kinase phosphorylation and impaired endothelial proliferation [41,42].

Another critical factor in defective angiogenesis is the altered expression of angiopoietins. Angiopoietin-1 (Ang-1), which promotes endothelial survival, is reduced in SSc, whereas angiopoietin-2 (Ang-2), a factor disrupting endothelial integrity, is significantly upregulated. This imbalance favors endothelial death and vascular regression rather than new vessel formation [43]. Furthermore, the friend leukemia virus integration 1 (FLI1) transcription factor, crucial for endothelial function, is epigenetically suppressed in SSc, contributing to aberrant endothelial behavior and defective vascular repair [44].

## 6. Endothelial to Mesenchymal Transition

Endothelial to mesenchymal transition (EndoMT) is increasingly recognized as a key contributor to the fibrotic vasculopathy observed in SSc. This transdifferentiation process involves endothelial cells losing their typical markers (e.g., vWF, CD31, and VE-cadherin) and acquiring mesenchymal features, including expression of α-smooth muscle actin (α-SMA), collagen type I, and SM22α [45]. In SSc, subendothelial accumulation of activated myofibroblasts has been documented in arterioles of the lung and kidney, with evidence that a subset of these cells derives from transformed endothelial cells via EndoMT [46].

Histological and in vitro studies support this phenomenon. Pulmonary vessels from SSc patients and animal models such as hypoxia/SU5416 mice demonstrate endothelial cells co-expressing both endothelial and mesenchymal markers, confirming partial or full EndoMT. Pro-inflammatory and pro-fibrotic cytokines, including IL-1β, TNF-α, and TGF-β1, drive this transition, promoting cytoskeletal reorganization and barrier dysfunction. Cells undergoing EndoMT lose junctional integrity, contributing to vascular leakage and tissue fibrosis [47].

In SSc-associated interstitial lung disease (ILD), CD31+/CD102+ endothelial cells simultaneously expressing mesenchymal transcripts have been isolated, while in skin lesions and bleomycin or uPAR-deficient mouse models, intermediate EndoMT stages have been observed [48,49]. Molecular pathways implicated in EndoMT include PI3K/Akt/mTOR and leukotriene B4 (LTB4)/BLT1 signaling [50]. Additionally, endothelin-1, Wnt3a, IFN-γ, oxidative stress, hypoxia, and various microRNAs contribute to the initiation and maintenance of EndoMT [45]. Therefore, endoMT may be considered as a pivotal mechanism linking vascular dysfunction, immune-mediated inflammation, and fibrosis in SSc and, subsequently, as a druggable target to limit vasculopathy and fibrotic progression in affected organs.

## 7. Endothelial Dysfunction in Systemic Sclerosis: A Mechanistic Perspective

In SSc, endothelial dysfunction is increasingly recognized not merely as a consequence but as a fundamental initiating event in the disease process. Several pathogenic stimuli—including oxidative stress, infectious agents, and immune-mediated mechanisms—may cause early endothelial damage, triggering pathophysiological events. This initial endothelial insult activates both the coagulation cascade and platelet aggregation, leading to localized microthrombi and setting the stage for progressive microvascular occlusion (Table 1) [2].

The hallmark of SSc vasculopathy is the intimal hyperplasia of small arterioles, which leads to progressive luminal narrowing and chronic tissue hypoxia. The resulting ischemia–reperfusion cycles determine the leakage of vasoactive and profibrotic mediators, exacerbating local tissue damage and perpetuating vascular remodeling. Among these mediators, hypoxia-induced pathways activate resident fibroblasts and promote extracellular matrix deposition within the vessel wall, contributing to increased vascular stiffness and sustained endothelial dysfunction [51].

Dysfunctional endothelium contributes to impaired perfusion and chronic ischemia and acts as a biological amplifier of fibrotic signaling. Several mechanisms have been proposed to explain this link: endothelial injury enhances vascular permeability and promotes the release of profibrotic mediators, including TGF-) and connective tissue growth factor (CTGF), which directly stimulate fibroblast activation and myofibroblast differentiation [52]. Apoptotic endothelial cells may also recruit inflammatory cells, particularly macrophages, that further enhance fibrogenic responses through paracrine cytokine release.

**Figure 1:**
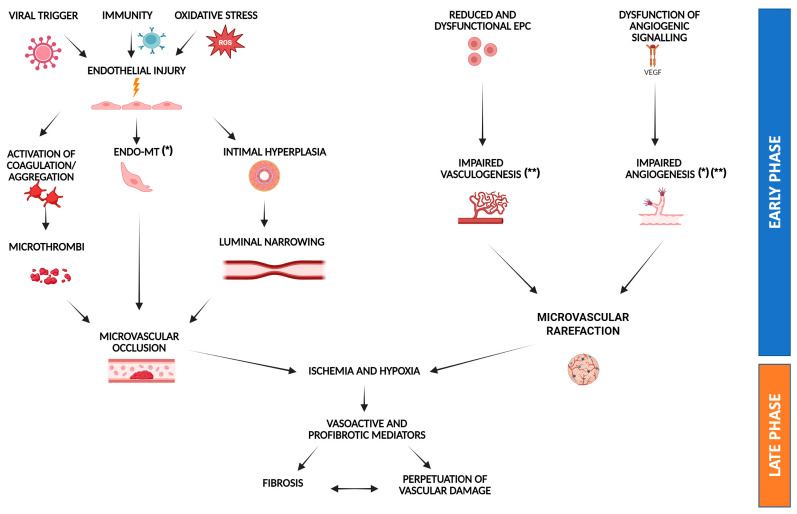
**Model of “fibrosing microangiopathy” in systemic sclerosis.** Schematic representation of the interconnected molecular and cellular mechanisms underlying endothelial dysfunction in SSc. Initial insults—including oxidative stress, viral infections, and immune-mediated endothelial cytotoxicity—induce endothelial activation and apoptosis, triggering microthrombosis, endothelial-to-mesenchymal transition (EndoMT), and intimal hyperplasia. These events culminate in progressive microvascular obliteration. Concurrently, defective vasculogenesis and angiogenesis—driven by quantitative and functional impairment of endothelial progenitor cells (EPCs), anti-angiogenic VEGF isoforms, angiopoietin system dysregulation, eNOS deficiency, and FLI1 downregulation—result in capillary rarefaction and chronic hypoxia. The ensuing release of profibrotic mediators, such as TGF-β and CTGF, fosters a self-perpetuating cycle of vascular injury and fibrosis. The figure highlights the progressive distinction between early (immune/oxidative) and late (fibrotic) events, and illustrates potential therapeutic intervention points within the pathogenic cascade. Potential molecular targets of emerging therapies are indicated by asterisks: (*) tyrosine kinase inhibitors; PDE4 inhibitors; sGC stimulators; (**) ROCK inhibitors.

This interplay is not SSc-specific: similar vasculo-fibrotic crosstalk is observed in other fibrosing disorders, such as idiopathic pulmonary fibrosis, where avascular fibrotic zones display failed neovascularization [53]. In SSc, however, vascular pathology often precedes fibrosis, offering a unique model to study this sequence. Notably, the fibrotic process, traditionally viewed as a downstream consequence of vascular damage, may itself contribute to the progression of vasculopathy. Myofibroblast infiltration into the vascular intima and media leads to concentric fibrosis, further impairing vascular compliance and perfusion. This reciprocal relationship between vascular injury and fibrotic remodeling supports the concept of SSc as a “fibrosing microangiopathy”, in which blood vessels are both primary targets and active contributors to disease progression (Figure 1).

A summary of key circulating endothelial biomarkers and their clinical implications in systemic sclerosis is provided in Table 2.

## 8. Clinical Manifestations and Therapeutic Strategies

The clinical spectrum of SSc highlights the pervasive impact of endothelial dysfunction across both microvascular and macrovascular territories. The most characteristic early feature is Raynaud’s phenomenon, a transient vasospastic response of the digital arteries, typically triggered by cold or emotional stress, resulting in triphasic color changes (pallor, cyanosis, and rubor), which is present in the vast majority of SSc patients. This episodic vasospastic response is rooted in the disruption of endothelial homeostasis, where diminished nitric oxide and prostacyclin signaling fails to counterbalance potent vasoconstrictors such as endothelin-1. The cumulative effect of this imbalance, exacerbated by oxidative stress and vascular wall remodeling, can transform a functional vasospasm into irreversible microvascular occlusion. Over time, this process contributes to digital ischemia and the formation of painful, non-healing ulcers affecting up to a third of patients annually [54].

Scleroderma renal crisis (SRC), though less common, represents a fulminant form of endothelial damage. It typically presents with abrupt hypertension, rapidly declining renal function, and signs of thrombotic microangiopathy. Histologically, the renal vasculature exhibits intimal thickening and lumen narrowing, compromising perfusion. Cytokines such as IL-6 have been implicated in SRC pathophysiology, directly linking immune dysregulation to vascular injury [55]. The upregulation of endothelin-1 and its receptors in the kidneys further reinforces the concept of a pathogenic vasculopathy responsive to targeted endothelin blockade [56].

PAH is another major vascular complication seen in a subset of SSc patients. It is characterized by increased pulmonary vascular resistance and right ventricular strain, often progressing insidiously. Aberrant endothelial signaling leads to reduced vasodilatory mediators and excessive vasoconstriction, while EndoMT and inflammatory processes foster vascular remodeling [57]. Markers such as IL-32 have emerged as potential biomarkers of disease activity in SSc-PAH [58].

Macrovascular involvement in SSc, though historically underappreciated, has garnered increasing attention. Evidence of premature atherosclerosis, coronary microvascular dysfunction, and large artery stiffness challenges the traditional view of SSc as a purely microvascular disease [59]. Non-invasive vascular imaging has demonstrated subclinical alterations in arterial structure and function, even in patients without overt cardiovascular risk factors. These changes correlate with an increased incidence of ischemic heart disease and may explain the high proportion of cardiovascular-related mortality. Despite this, formal guidelines for cardiovascular risk assessment in SSc are still lacking [2].

Therapeutic strategies targeting early vascular injury may thus offer an opportunity to intercept downstream tissue fibrosis progression before irreversible organ damage ensues. Current treatments of endothelial dysfunction include vasodilators such as prostacyclin analogs and phosphodiesterase-5 inhibitors, which improve perfusion and reduce ischemic injury. Endothelin receptor antagonists are not only beneficial to PAH but may also prevent digital ulcers. Emerging approaches include tyrosine kinase inhibitors, Rho/ROCK pathway modulators, and leukotriene B4 antagonists, which target vascular inflammation and remodeling. Moreover, mesenchymal stem cell therapy and immunomodulators such as rituximab are under investigation to restore vascular integrity and attenuate fibrosis. These strategies reflect a growing therapeutic focus on halting the vascular-fibrotic continuum at its source [2]. Strategies to restore endothelial homeostasis and prevent fibrotic progression—ranging from vasodilators to emerging immunomodulatory and antifibrotic agents—may prove beneficial in modifying disease trajectory and improving long-term outcomes of SSc patients. For instance, the antifibrotic potential of phosphodiesterase 4 (PDE4) inhibitors, such as nerandomilast, is gaining attention. Exhibiting a preferential inhibition of PDE4B, nerandomilast showed anti-inflammatory and antifibrotic effects in preclinical models and demonstrated efficacy in modulating key parameters of lung fibrosis in both bleomycin and silica-induced mouse models [60]. In vitro, this novel agent inhibits myofibroblast transformation and extracellular matrix protein expression [61]. Interestingly, nerandomilast also significantly reduced microvascular permeability in cytokine-stimulated human lung microvascular endothelial cells [61]. Furthermore, NGS data of a bleomycin mouse model confirmed the beneficial effect of nerandomilast on markers of vascular dysfunction in vivo [62]. Consistent with these findings, Hamada et al. elegantly showed that in the bleomycin mouse model, PDE4B was preferentially expressed in non-immune cells such as capillary endothelial cells, fibroblasts, and alveolar type2 cells. Importantly, epithelial cell apoptosis was reduced, and regenerated epithelial cells were found in nerandomilast-treated mice [63]. Taken together, these findings position nerandomilast as a promising candidate for the treatment of SSc, where vascular dysfunction, inflammation, and fibrosis contribute to disease progression.

## 9. Conclusions

SSc is a paradigmatic disease in which endothelial dysfunction plays a central, initiating role in the pathogenesis rather than being a mere downstream effect of autoimmunity and fibrosis. The multifactorial nature of endothelial injury—driven by exogenous triggers, inflammatory immune-mediated responses, and defective vascular repair—leads to chronic microvascular and macrovascular damage. This vascular pathology underlies hallmark clinical manifestations such as Raynaud’s phenomenon, digital ulcers, pulmonary arterial hypertension, and scleroderma renal crisis. Mechanistically, impaired angiogenesis and vasculogenesis, along with processes like EndoMT, contribute to progressive ischemia and fibrotic vascular and tissue remodeling. These insights reinforce the concept of SSc as a model of systemic endothelial dysfunction and highlight the therapeutic potential of early targeting of vascular injury. Strategies to restore endothelial homeostasis and prevent fibrotic progression—ranging from vasodilators to emerging immunomodulatory and antifibrotic agents—may prove beneficial in modifying disease trajectory and improving long-term outcomes of SSc patients.

## Figures and Tables

**Table 1:** Mechanisms of endothelial dysfunction in systemic sclerosis (SSc).

Mechanism	Molecular/Cellular Factors Involved	Pathological Consequences
Immune-mediated mechanism	CD8+ cytotoxic T cells, anti-endothelial cell antibodies (AECAs), HLA class I/II expression	Endothelial activation and apoptosis, increased IL-6, ICAM-1, TGF-β1, fibrosis, vasoconstriction
Oxidative stress	Reactive oxygen species (ROS), decreased nitric oxide (NO), ischemia–reperfusion injury	Vasoconstriction, platelet aggregation, endothelial apoptosis
Viral infections	HCMV (UL94), EBV (TLR9, IFN pathways), parvovirus B19	Endothelial cytotoxicity, apoptosis, inflammation
Impaired vasculogenesis	Decreased EPCs (CD34+ CD133+ VEGFR2+), bone marrow fibrosis, pentraxin-3, Akt-FOXO3a-Bim pathway	Reduced vascular repair, digital ulcers, pulmonary arterial hypertension
Impaired angiogenesis	eNOS deficiency, VEGF165b (antiangiogenic isoform), reduced Ang-1, increased Ang-2, FLI1 deficiency	Capillary rarefaction, chronic hypoxia, endothelial dysfunction
Endothelial-to-mesenchymal transition (EndoMT)	TGF-β1, endothelin-1, Wnt3a, hypoxia, microRNAs, IL-1β, TNF-α, oxidative stress, PI3K/Akt/mTOR, LTB4/BLT1	Myofibroblast differentiation, vascular remodeling, fibrosis
Pro-thrombotic state and vascular remodeling	Coagulation cascade activation, platelet aggregation, CTGF, chronic hypoxia	Intimal hyperplasia, luminal narrowing, ischemia, fibrosis

**Table 2:** Key circulating endothelial biomarkers and their clinical relevance in SSc.

Biomarker	Biological Role	Alteration in SSc	Clinical Relevance
ET-1	Potent vasoconstrictor; promotes fibrosis and vascular remodeling	Elevated in serum and tissues	Correlates with severity of vasculopathy, digital ulcers, PAH, and renal crisis
VCAM-1	Promotes leukocyte adhesion to activated endothelium	Increased expression on endothelial cells and in circulation	Marker of endothelial activation; associated with inflammation and early microvascular damage
vWF	Mediates platelet adhesion; marker of endothelial injury	Elevated plasma levels	Indicator of endothelial damage and thrombotic risk; associated with PAH and digital ulcers
IL-6	Pro-inflammatory cytokine; promotes acute-phase response	Elevated serum levels	Predicts disease progression, vascular complications (e.g., SRC, PAH), and fibrosis
sJAM-1	Maintains endothelial integrity; regulates leukocyte transmigration	Increased in circulation	Associated with endothelial disruption; elevated in early SSc and vasculopathic complications
E-selectin	Facilitates leukocyte rolling on activated endothelium	Elevated serum levels	Reflects endothelial activation; linked to inflammation and Raynaud’s phenomenon
Ang-2	Antagonist of Ang-1; disrupts endothelial stability	Elevated in serum	Associated with vascular regression and disease severity; predictor of PAH
VEGF	Stimulates angiogenesis	Elevated, especially antiangiogenic VEGF165b isoform	Paradoxical role: increased levels yet impaired angiogenesis; related to capillary rarefaction
NO	Vasodilator; maintains vascular tone and inhibits platelet aggregation	Reduced bioavailability	Contributes to vasoconstriction, endothelial dysfunction, and impaired tissue perfusion
TGF-β	Profibrotic cytokine; regulates immune responses and extracellular matrix production	Overexpressed in affected tissues	Central mediator of fibrosis and vasculopathy; therapeutic target in SSc
TNF-α	Tumor necrosis factor-alpha	Pro-inflammatory cytokine; enhances endothelial activation, leukocyte adhesion, and perpetuates vascular inflammation	Promotes chronic inflammation and endothelial activation; associated with digital ulcers and fibrotic progression in SSc

## Data Availability

No new data was generated for this narrative review.

## Abbreviations

The following abbreviations are used in this manuscript:

α-SMA	α-smooth muscle actin
AECAs	Autoantibodies directed against endothelial cell antigens
Ang-1 and 2	Angiopoietin-1 and 2
CCL2	C-C motif chemokine ligand 2
CTGF	Connective tissue growth factor
EBV	Epstein–Barr virus
eNOS	Endothelial nitric oxide synthase
EndoMT	Endothelial to mesenchymal transition
EPCs	Endothelial progenitor cells
ET-1	Endothelin-1
FGF-2	Fibroblast growth factor-2
FLI1	Friend leukemia virus integration 1
FMD	Flow-mediated dilation
HCMV	Human cytomegalovirus
HLA	*Human Leukocyte Antigen*
ICAM-1	Intercellular adhesion molecule-1
IL-6	Interleukin-6
ILD	Interstitial lung disease
IMT	Intima-media thickness
LTB4	Leukotriene B4
NO	Nitric oxide
PAH	Pulmonary arterial hypertension
PDE4	Phosphodiesterase 4
PDGF	Platelet-derived growth factor
ROS	Reactive oxygen species
SDF-1	Stromal cell-derived factor-1
SRC	Scleroderma renal crisis
TGF-β	Transforming growth factor-beta
TLR9	Toll-like receptor 9
TNF-α	Tumor necrosis factor-alpha
sJAM-1	Soluble Junctional Adhesion Molecule-1
SSc	Systemic sclerosis
VCAM-1	Vascular cell adhesion molecule-1
VEGF	Vascular endothelial growth factor
VEGF165b	Vascular Endothelial Growth Factor 165b
VEGFR2	Vascular endothelial growth factor receptor 2
vWF	Von Willebrand factor
